# Relationship between paternal smoking behaviour and birth outcomes based on a comic booklet intervention for preventing second-hand smoke exposure to non-smoking pregnant women in Indonesia: a follow-up randomised controlled trial

**DOI:** 10.1186/s41182-025-00701-z

**Published:** 2025-02-19

**Authors:** Kimiko Inaoka, Citra Gabriella Mamahit, Ishak Halim Octawijaya, Windy Mariane Virenia Wariki, Erika Ota

**Affiliations:** 1 Global Health Nursing, The School of Nursing Science, International University of Health and Welfare, Narita, Japan; 2 Global Health Nursing, Graduate School of Nursing Science, St. Luke’s International University, Tokyo, Japan; 3 Global Public Health Department, Graduate School of Comprehensive Human Sciences, University of Tsukuba, Tsukuba, Japan; 4 School of Nutrition and Dietetics, Faculty of Health and Social Services, Kanagawa University of Human Services, Yokosuka, Japan; 5 Department of Community Medicine, Faculty of Medicine, Sam Ratulangi University, Manado, Indonesia

**Keywords:** Birth outcome, Paternal behaviour, Pregnant women, Randomised controlled trial, Second-hand smoke

## Abstract

**Background:**

Although the harmfulness of second-hand smoke (SHS) exposure to foetuses is well-established, literature reporting foetal outcomes in experimental studies is limited. This follow-up study on preventing SHS exposure among non-smoking Indonesian pregnant women at home was based on a randomised controlled trial involving the provision of comic booklets with stickers to couples. This trial examined differences in the birth outcomes of participating couples between the experimental and control groups, factors associated with paternal smoking behaviour, and association between birth outcomes and paternal-related outcomes.

**Methods:**

In total, 197 neonates of 286 couples who participated in an original trial were included. This study compared birth outcomes between participating couples using a comic booklet with stickers to reduce SHS exposure at home during pregnancy as the intervention. Pearson Chi-square tests were conducted to investigate significant differences in neonate sexes between the experimental and control groups. Independent sample t-tests were used to check for significant differences in birth outcome data between the experimental and control groups. A multiple regression analysis was applied to test the correlation between paternal smoking behaviour and the birth outcomes.

**Results:**

The gestational age in the experimental group was longer than the age in control group (mean difference = 0.373, Cohen’s d = 0.291, 95% CI [0.010–0.57], p-value = 0.048). Pregnant women’s avoidance of SHS strongly influenced paternal smoking behaviour in both the experimental group (*b* = 0.559, 95% CI [1.175–2.109], *p*-value < 0.001) and the control group (*b* = 0.429, 95% CI [0.675–1.567], *p*-value < 0.001). No associations were observed between birth and paternal behaviour outcomes.

**Conclusions:**

The neonates’ gestational ages were greater in the experimental group than in the control group because of our intervention effect; pregnant women’s avoidance of SHS strongly influenced paternal smoking behaviour in both groups. Thus, the comic booklet intervention for smoking fathers with non-smoking pregnant partners helped reduce the risk of foetal developmental disorders. Couple-based interventions should be actively integrated into health worker strategies to effectively mitigate second-hand smoke exposure among pregnant women.

*Trial registration* This study was registered in the UMIN Clinical Trials Registry under the registration number UMIN000035423 (01/02/2019).

## Background

The foetal developmental environment affects the risks for postnatal diseases [1]. Second-hand smoke (SHS) exposure in foetuses at birth is associated with risks of stillbirth [2], congenital malformation [2, 3], low birth weight [4, 5], smaller head circumference [5], shorter length [4], and younger gestational age [6]. Particularly, paternal smoking poses a health risk to neonates [7, 8].

Few interventional studies have investigated the effect of smoking trends on the offspring of people who smoke. Nwosu et al. [9] conducted a systematic review of nine studies, only one study involved smoking partners, and found that multi-component interventions were effective. Pollak et al. [10] conducted a couple-based randomised controlled trial (RCT) on the effects quitting paternal smoking during the perinatal and the postpartum period using counselling, and demonstrated no arm differences in smoking rates at the endpoint. Inaoka et al. [11] conducted a couple-based RCT (using comic booklets and reminders) following the Health Belief Model (HBM) to reduce SHS exposure at home during pregnancy, and reported that appropriate smoking behaviours were recognised in the experimental group (EG) by fathers who smoked.

There is a dearth of literature reporting foetal outcomes in experimental studies. El-Mohandes et al.[12] conducted an RCT on decreasing SHS exposure among non-smoking pregnant women from smokers in their home, room, or car, and found a low incidence of very low birth weight and preterm birth in the EG. However, the association between paternal smoking behaviour and birth outcomes has not been investigated in trials.

The purposes of the study were: (1) to investigate differences in birth outcomes between participating couples in the EG and control group (CG) in an RCT [11]; (2) to examine paternal smoking behaviour, pregnant women’s avoidance of SHS exposure, and paternal health beliefs; and (3) to examine whether poor birth outcomes were associated with paternal smoking behaviour.

## Methods

### Study design

#### Original study

An RCT [11] examined the effectiveness of a comic booklet intervention for preventing SHS in non-smoking pregnant women among 348 couples who visited public health facilities (health centres and posts) for their first antenatal care appointment and were recruited at two towns in North Sulawesi, Indonesia, from March 2019 to March 2020. Of these, 62 couples were excluded for various reasons, such as not meeting the inclusion criteria or declining to participate. The 286 admitted pairs were assigned to the EG (140 couples) or CG (146 couples) using a central randomisation procedure. Additionally, the participants were randomly allocated to the two groups that received an intervention involving a comic booklet with stickers (EG) or usual care (CG).

A printed full-colour educational comic booklet with stickers was provided to the participating couples assigned to the EG. The comic booklet included eight sections that utilised behavioural change techniques [13, 14] and components of the HBM [15–17].

**Fig. 1:**
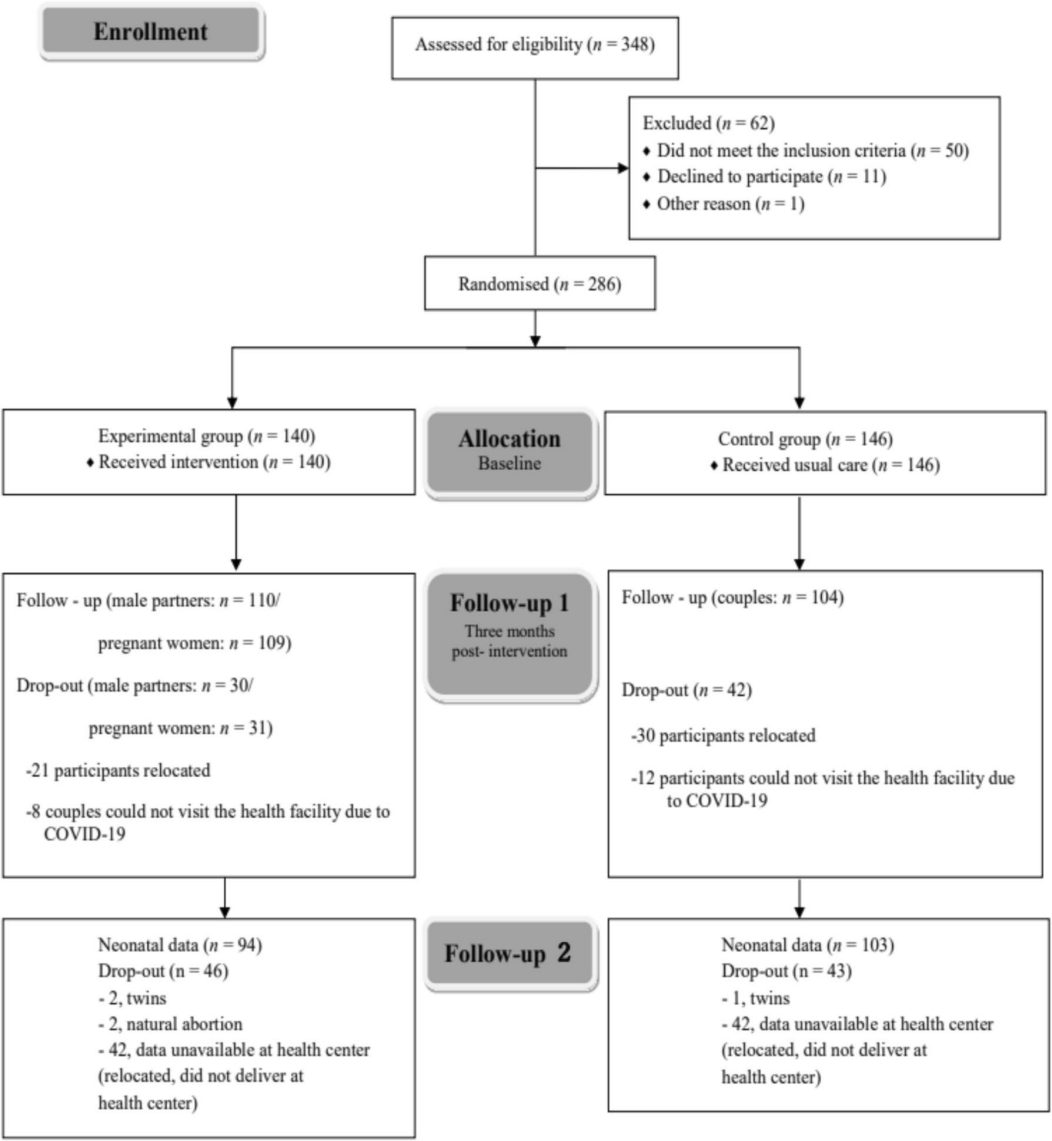
Flow diagram of participant selection

Paternal smoking behaviours and pregnant women’s avoidance of SHS exposure were evaluated at baseline and 3 months post-intervention (baseline and follow-up 1, respectively, in Fig. 1). Data collection for follow-up 1 was completed by the end of October 2020.

Measurements of father’s outcomes have been reported in the methods section of this study. Further details about the original study methods have been published elsewhere [11].

#### This follow-up study

This follow-up study of the RCT examined (1) differences in the birth outcomes of the participating couples between the EG and CG, (2) factors associated with paternal smoking behaviour collected at follow-up 1 at 3 months post-intervention, and (3) the association between poor birth outcomes and paternal smoking behaviour.

### Participants

The participants in this follow-up study were neonates of couples who participated in the original RCT. Their fathers’ primary and secondary outcomes from the original RCT were used. Inaoka et al. [11] detailed the inclusion criteria, participant flow, characteristics, and sample size calculation in their study.

### Measures

For fathers, the primary outcome of the original RCT was smoking-related behavioural responses in the proximity of their pregnant partners. The questionnaire contained paternal self-report of smoking behaviours at home (A) and the pregnant partner’s avoidance of SHS (B). The questionnaire contained 11 items. They scored their level of agreement on a four-point Likert scale ranging from 1 to 4, with higher values indicating more appropriate smoking behaviours of male partners [11].

The secondary outcomes were SHS knowledge (C), health beliefs based on the HBM (D–H), and self-efficacy (I) assessed through paternal self-report questionnaires for couples. The questionnaire comprised 40 items. For SHS knowledge, fathers were asked to select either 'yes' or 'no' for each question. Correct and incorrect responses received 1 and 0 points, respectively. For health beliefs and self-efficacy, participants rated their level of agreement with each statement on a four-point Likert scale ranging from 1 to 4, with higher values indicating more appropriate health beliefs and greater self-efficacy. Lower values indicated more appropriate health beliefs for perceived barriers [11].

The General Self-Efficacy Scale (GSES) [18] was used, and its reliability was tested. Alphas ranged from 0.82 to 0.93 among German participants in 1989 (p.35) [19]. Concurrent and predictive validities were assessed for the GSES (p.36). Furthermore, the retest reliability was 0.47 and 0.63 for men and women, respectively, in 1991 (p.36) [19].

The neonates’ sex, birth weight, birth height, and gestational age at delivery, which were obtained at the health facilities in Indonesia, were used as indicators for the effects of environmental smoking on the foetuses. Specifically, the collection of neonates’ outcomes was completed in November 2022.

The neonates’ family characteristics (i.e. type of family and exposure to SHS from family smokers) were obtained at baseline.

In particular, the self-report questionnaire, except for self-efficacy, was initially designed in English by K.I. with guidance from E.O., which was subsequently translated into Indonesian with the assistance of Indonesian researchers. The questionnaire was also independently back-translated into English to verify the quality of the translation before being used for field implementation. Moreover, the Indonesian adaptation of the GSES has been translated into Indonesian by Born, Schwarzer, and Jerusalem [20].

### Data analyses

Data were analysed using the IBM Statistical Package for the Social Sciences (SPSS), version 29, for Windows.

To explore differences in the neonate birth outcomes of the couples in the EG and CG, the comic booklet intervention was used as the primary independent variable. First, the missing completely at random (MCAR) test [21, 22] was performed for all birth outcome data in the two arms. Second, Pearson Chi-square tests were conducted, based on the assumptions of the central limit theorem [20], to investigate significant differences in neonates’ sex, type of family, and exposure to SHS from family smokers between the EG and CG. Independent sample *t*-tests (two-tailed) were used to check for significant differences in birth outcome data (birth weight, height, and gestational age) between the EG and CG, without checking for normality based on the central limit theorem assumptions [23]. A 95% confidence interval (CI) value (*p* < 0.05) was considered statistically significant. The effect sizes were estimated and evaluated using Cohen’s d [24, 25].

To explore whether paternal smoking behaviour was associated with pregnant women’s avoidance of SHS exposure and paternal health beliefs, the independent variables used were the paternal-evaluated avoidance of SHS exposure by the pregnant women, and the self-evaluated paternal health beliefs (knowledge, disease susceptibility, disease severity, benefits, barriers, cues to actions, and self-efficacy). Pearson product–moment correlation coefficient was applied to determine the correlation among the independent variables (paternal-related variables). A multiple regression analysis (MRA) was applied to test the correlation between paternal smoking behaviour and independent variables that were unconfirmed as MCAR (*p*-value ≤ 0.01) 3 months post-intervention [11].

To explore whether birth outcomes were associated with paternal outcomes, the independent variables used were paternal smoking behaviour, pregnant women’s avoidance of SHS exposure, and self-evaluated paternal health beliefs (knowledge, disease susceptibility, disease severity, benefits, barriers, cues to action, and self-efficacy). The MRA was applied to test the correlation between the birth outcomes and paternal outcomes that were unconfirmed as MCAR (*p*-value ≤ 0.01) 3 months post-intervention [11].

## Results

### Participants

Figure 1 displays the flow diagram of the participant selection process. For the baseline analysis, of the 348 pairs who agreed to join, data from 286 couples who filled the inclusion criteria were analysed. At 3 months post-intervention, 110 couples’ fathers (79% response rate, 21% dropout rate) in the EG and 104 couples (71% response rate, 29% dropout rate) in the CG provided data for the primary and secondary outcomes. The final number of couples was 214 (EG 110; CG 104); reasons for dropping out at 3 months post-intervention included relocation or inability to visit the health facility during the COVID-19 pandemic. In all, 197 neonates were included in this study: 94 neonates (67% response rate, 33% dropout rate) in the EG and 103 (71% response rate, 29% dropout rate) in the CG. Thus, completion of this follow-up study was delayed for approximately 1 year due to the COVID-19 pandemic.

### Neonate and family characteristics

The paternal characteristics did not differ between the groups [11]. Table 1 lists the neonate characteristics and the family characteristics of neonates. No significant differences were observed between the groups (sex: *p*-value = 0.362, type of family: *p*-value = 0.583, and exposure to SHS from family smokers: *p*-value = 0.155).

**Table 1:** Neonates and their family characteristics

	n	EG n (%)	CG n (%)	*φ*	*p*-value
Boy	101	45 (47.872)	56 (53.846)	− 0.065	0.362^a^
Girl	96	49 (52.128)	47 (45.192)		
Types of family					0.583^a^
Nuclear family	142	72 (51.4)	70 (47.9)		
Joint family	135	64 (45.7)	71 (48.6)		
N.A	9	4 (2.9)	5 (3.4)		
Exposure to SHS from family smokers at home	66	27 (19.3)	39 (26.7)		0.155^a^

EG: experimental group, CG: control group, SHS: second-hand smoke

^a^Chi-square test was conducted

### Birth outcome analysis (Table 2)

**Table 2:** Differences in birth weight, birth height, and gestational age between the EG and CG

	EG (*n* = 94)	CG (*n* = 103)	*MD*	*Cohen’sd*	p-value	*95% CI*
	*Mean* (SD)	*Mean* (SD)				
Birth weight (g)	3065.200 (371.383)	3037.070 (353.006)	28.132	0.077	0.589^a^	− 0.202, 0.357
Birth height (cm)	48.126 (2.378)	48.216 (2.192)	− 0.091	− 0.040	0.785^a^	− 0.319, 0.240
Gestational age (week)	39.362 (1.335)	38.989 (1.230)	0.373	0.291	0.048^a^	0.010, 0.57

EG: experimental group, CG: control group; *MD*: mean difference; *CI*: confidence interval

^a^t-test was conducted

For all birth outcomes, MCAR was not confirmed (p-value ≤ 0.001) because the significance level was p < 0.05.

Birth weight (Cohen’s *d* = 0.077, 95% CI [− 0.202 to 0.357], *p*-value = 0.589) and height (Cohen’s d = − 0.040, 95% CI [− 0.319 to 0.240], *p*-value = 0.785) did not differ between the groups. However, gestational age was significantly longer in the intervention group (39.36 ± 1.34 weeks) than in the control group (38.99 ± 1.23 weeks), with a small effect size (Cohen’s *d* = 0.291, 95% CI [0.010–0.57], *p* = 0.048).

### Association between the paternal smoking behaviour and independent variables (pregnant woman’s avoidance of SHS exposure and paternal health beliefs)

In the previous study, MCAR was not confirmed for paternal-related variables at 3 months post-intervention (*p*-value ≤ 0.01). The correlation among the independent variables (paternal-related variables) was not strong (*r* > 0.9) (Tables 3 and 4). Two variables were fairly correlated in both groups: (1) paternal smoking behaviour and pregnant woman’s avoidance of SHS (EG: *r* = 0.64; CG: *r* = 0.59); and (2) disease susceptibility and disease severity (EG *r* = 0.60, CG *r* = 0.56).

**Table 3:** Correlation coefficients between independent variables in the EG (*n* = 110)

Independent variables	A	B	C	D	E	F	G	H	I
A. Paternal smoking behaviour	–	0.64**	0.28**	0.33**	0.33**	0.21*	− 0.03	0.41**	0.30**
B. Pregnant women’s avoidance of SHS	0.64**	–	0.15	0.43**	0.34**	0.25**	0.16	0.25**	0.16
C. Knowledge of SHS	0.28**	0.15	–	0.11	0.19	0.14	0.04	0.12	0.23**
D. Perceived SHS-related disease susceptibility	0.33**	0.43**	0.11	–	0.60**	0.45**	− 0.02	0.28**	0.50**
E. Perceived SHS-related disease severity	0.33**	0.34**	0.19	0.60**	–	0.17	− 0.05	0.31**	0.48**
F. Perceived benefits	0.21*	0.25**	0.14	0.45**	0.17	–	0.05	− 0.17	0.13
G. Barriers to preventing SHS exposure	− 0.03	0.16	0.04	− 0.02	− 0.05	0.05	–	− 0.11	− 0.12
H. Cue to action for preventing SHS exposure	0.41**	0.25**	0.12	0.28**	0.31**	− 0.17	− 0.11	–	0.47**
I. Self-efficacy	0.30**	0.16	0.23**	0.50**	0.48**	0.13**	− 0.12	0.47**	–

SHS: second-hand smoking

*p < 0.05, **p < 0.01

**Table 4:** Correlation coefficients for the independent variables in the CG (*n* = 104)

Independent variables	A	B	C	D	E	F	G	H	I
A. Paternal smoking behaviour	–	0.59**	0.42**	0.38**	0.29**	0.08	0.01	0.42**	0.20*
B. Pregnant women’s avoidance of SHS	0.59**	–	0.29**	0.39**	0.32**	0.09	− 0.07	0.26**	0.22*
C. Knowledge of SHS	0.42**	0.29**	–	0.49**	0.34**	0.16	− 0.03	0.13	0.03
D. Perceived SHS-related disease susceptibility	0.38**	0.39**	0.49**	–	0.56**	0.40**	0.05	0.25*	0.01
E. Perceived SHS-related disease severity	0.29**	0.32**	0.34**	0.56**	–	0.20*	− 0.11	0.43**	0.23*
F. Perceived benefits	0.08	0.09	0.16	0.40**	0.20*	–	0.05	0.14	0.12
G. Barriers to preventing SHS exposure	0.01	− 0.07	− 0.03	0.05	− 0.11	0.05	–	− 0.06	− 0.30**
H. Cue to action for preventing SHS exposure	0.42**	0.26**	0.13	0.25*	0.43**	0.14	− 0.06	–	0.32**
I. Self-efficacy	0.20*	0.22*	0.03	0.01	0.23*	0.12	− 0.30**	0.32**	–

SHS: second-hand smoke, *p < 0.05, **p < 0.01

The multiple regression model showed a good fit for the EG (*R* = 0.73, *R*^2^ = 0.53, adjusted *R*^2^ = 0.50) (Table 5) due to the paternal smoking behaviour being strongly influenced by the pregnant woman’s avoidance of SHS (*b* = 0.559, 95% CI [1.175–2.109], *t*-value = 6.897, *p*-value < 0.001), cue to action to prevent SHS exposure (*b* = 0.262, 95% CI [0.080–0.362], *t*-value = 3.074, *p*-value = 0.002), and knowledge of SHS (*b* = 0.143, 95% CI [0.004–1.265], *t*-value = 1.973, *p*-value = 0.049). Multicollinearity was not observed.

**Table 5:** Estimated standardised regression coefficients for self-reported paternal smoking behaviour in the EG (*n* = 109)

Variables	Standardised regression coefficients beta (b)	*t-value*	*95% CI*
B. Pregnant woman’s avoidance of SHS	0.559	6.897	1.175, 2.109
C. Knowledge of SHS	0.143	1.973	0.004, 1.265
D. Perceived SHS-related disease susceptibility	− 0.132	− 1.236	− 1.679, 0.381
E. Perceived SHS-related disease severity	0.057	0.618	− 0.513, 0.985
F. Perceived benefits	0.143	1.703	− 0.038, 0.537
G. Barriers to preventing SHS exposure	− 0.096	− 1.375	− 0.373, 0.065
H. Cue to action for preventing SHS exposure	0.262	3.074	0.080, 0.362
I. Self-efficacy	0.062	0.653	− 0.141, 0.281

*R* = 0.73, *R*^2^ = 0.53, adjusted *R*^2^ = 0.50, ANOVA *p* < 001, Durbin–Watson = 1.69

Rounded to the fourth decimal place. SHS: second-hand smoke, *CI*: confidence interval, ANOVA: analysis of variance

The multiple regression model was a good fit for the CG (*R* = 0.70, *R*^2^ = 0.50, adjusted *R*^2^ = 0.45) (Table 6). The MRA indicated that pregnant women’s avoidance of SHS (*b* = 0.429, 95% CI [0.675–1.567], *t*-value = 4.939, *p*-value < 0.001) was a strong influence for fathers’, whereas cue to action and knowledge of SHS exerted a weak influence (*b* = 0.292, 95% CI [0.102–0.363], *t*-value = 3.481, *p*-value < 0.001; *b* = 0.256, 95% CI [0.298–1.596], *t*-value = 2.868, *p*-value = 0.004). Multicollinearity was not observed.

**Table 6:** Estimated standardised regression coefficients for self-reported paternal smoking behaviour in the CG (*n* = 103)

Variables	Standardised regression coefficients beta (b)	*t-value*	*95% CI*
B. Pregnant women’s avoidance of SHS	0.429	4.939	0.675, 1.567
C. Knowledge of SHS	0.256	2.868	0.298, 1.596
D. Perceived SHS-related disease susceptibility	0.092	0.867	− 0.557, 1.442
E. Perceived SHS-related disease severity	− 0.103	− 1.066	− 1.378, 0.407
F. Perceived benefits	− 0.065	− 0.798	− 0.545, 0.230
G. Barriers to preventing SHS exposure	0.074	0.959	− 0.171, 0.498
H. Cue to action for preventing SHS exposure	0.292	3.481	0.102, 0.363
I. Self-efficacy	0.063	0.756	− 0.112, 0.252

*R* = 0.70, *R*^2^ = 0.50, adjusted *R*^2^ = 0.45, ANOVA *p* < 0.001, Durbin–Watson = 2.13

Rounded to the fourth decimal place. SHS: second-hand smoke, *CI*: confidence interval, ANOVA: analysis of variance

### Association between birth outcomes and paternal-related variables

The results showed no confirmed statistical differences in all birth outcomes including birth weight (EG: *R* = 0.206, *R*^2^ = 0.043, adjusted *R*^2^ = − 0.071, analysis of variance [ANOVA] *p* = 0.915, Durbin–Watson = 1.950; CG: *R* = 0.247, *R*^2^ = 0.061, adjusted *R*^2^ = − 0.042, ANOVA *p* = 0.783, Durbin–Watson = 1.704), birth height (EG: *R* = 0.374, *R*^2^ = 0.140, adjusted *R*^2^ = 0.038, ANOVA *p* = 0.238, Durbin–Watson = 2.037; CG: *R* = 0.261, *R*^2^ = 0.069, adjusted *R*^2^ = − 0.033, ANOVA *p* = 0.713, Durbin–Watson = 1.789), and gestational age (EG: *R* = 0.194, *R*^2^ = 0.039, adjusted *R*^2^ = − 0.076, ANOVA p = 0.938, Durbin–Watson = 1.965; CG: *R* = 0.310, *R*^2^ = 0.096, adjusted *R*^2^ = − 0.003, ANOVA *p* = 0.468, Durbin–Watson = 1.925).

## Discussion

### Key results

This RCT yielded three important findings [11]: (1) gestational age of the EG was significantly greater than the age of CG; (2) pregnant women’s avoidance of SHS had a strong influence on paternal smoking behaviour in both groups; (3) birth outcomes such as birth weight, birth height, and gestational age were not associated with paternal-related variables; and (4) to the best of our knowledge, this was the first RCT to establish an association between paternal smoking behaviour and birth outcomes.

Birth outcomes, particularly shorter birth length [26], lower birth weight, [5, 27], and decreased gestational age [27], were associated with paternal smoking. In contrast with the results of previous studies [27], in this study, gestational age had a small effect on infant size, while paternal smoking behaviour and health beliefs had an impact on gestational age. Our results indicated that a comic booklet intervention with stickers (reminder) was effective for improving paternal smoking behaviour [11] at 3 months post-intervention. Meillier et al. [28] demonstrated that the process of changing health behaviour for men involves five steps: (1) time of interest; (2) time to process knowledge; (3) motivation arising from a cue to action; (4) new cue to stimulate action; and (5) reminders to maintain health habit. Herein, the first four steps had been conducted using a comic booklet. The fifth step was performed using stickers as a reminder to maintain the paternal new habit. Then, our intervention study aimed to confirm that the promoted smoking cessation behaviour among fathers prevented exposure of the foetus to SHS [11]. However, approximately 45.7% of couples in the experimental group at baseline were part of a joint family and living with smoking family members (42.2% of joint family in EG) other than smoking fathers. It is possible that the smoking behaviour of family members other than the father may have affected birth outcomes. A novelty of this interventional study is that it investigated the effect of avoiding SHS on birth outcomes. Previous studies based on the HBM did not evaluate this [29–32].

Briefly, ‘Knowledge of SHS’, refers to knowledge of the harmful nature of tobacco for pregnant women and foetuses, toxic substances contained in smoke, and the impact of indoor smoking and how it can indirectly influence behaviours [17]. ‘Cue to action for preventing SHS exposure’ indicated awareness of SHS risks, advice, and stickers for preventing SHS through perceived threats formed by the pairing of susceptibility and severity [17]. Furthermore, we verified the strong influence of ‘pregnant women’s avoidance of SHS’ and weak influence of ‘cue to action for preventing SHS exposure’ and ‘knowledge of SHS’ in both groups. This finding was partly consistent with those of previous studies. Martire et al.[33] showed the effectiveness of couple-based interventions for health behaviour; pregnant women’s avoidance of SHS influenced their male partners’ smoking behaviour. Furthermore, HBM components were linked to each other and health behaviours [17]. This interaction between paternal smoking behaviour and independent variables was not confirmed by previous studies, which is another novelty of our study [29–32].

### Study implications

The comic booklet intervention helped reduce the risk of foetal developmental disorders; further, the use of infographics as teaching material can be applied to various health education topics including pregnancy, childbirth, adult health, and disease prevention. Such interventions enhance the understanding of those interested in engaging in health education and behavioural change. In addition, health workers should consider employing couple-based interventions for accelerating educational effectiveness.

### Limitations

This study had several limitations. First, an intention-to-treat analysis, which reduces bias in construing a research’s results, could not be conducted because birth outcomes were not gathered timely from all participants at follow-up owing to some participants moving from the research location and COVID-19 restrictions. Therefore, the sample size was smaller than the original target. The dropout rates were high for both the EG (33%) and CG (29%). However, the participants provided similar reasons (e.g., relocation, restriction on visits to health facilities due to COVID-19) for dropping out in both groups, indicating low attrition bias. Finally, the randomisation approach lacked participant and evaluator blinding, although this did not affect the outcomes.

## Conclusions

This RCT yielded three important findings [11]: (1) the neonate gestational ages in the EG were longer than that in the CG, indicating the effect of this intervention, as noted in a previous study [27]; (2) pregnant women’s avoidance of SHS had a strong influence on the paternal smoking behaviour in both groups; and (3) birth outcomes were not related to paternal-related variables.

To address the effect size, it was more constructive to study interventions that reinforce smoking segregation in households and communities than employing a direct approach to the persistent smoking culture in Indonesia. A higher number of increased smoke-free areas is important for pregnant women and children along with fathers who smoke and do not want their families exposed to passive smoking.

Our study demonstrated the effectiveness of a couple-oriented comic booklet intervention in reducing the risk of foetal developmental disorders.

## Data Availability

No datasets were generated or analysed during the current study.

## Abbreviations

CG: Control group
EG: Experimental group
GSES: General Self-Efficacy Scale
HBM: Health Belief Model
MCAR: Missing completely at random
MRA: Multiple regression analysis
RCT: Randomised controlled trial
SHS: Second-hand smoke
